# AdhMMP8 Vector Administration in Muscle: An Alternate Strategy to Regress Hepatic Fibrosis

**DOI:** 10.3390/cells12172127

**Published:** 2023-08-22

**Authors:** Jesús García-Bañuelos, Edén Oceguera-Contreras, Ana Sandoval-Rodríguez, Blanca Estela Bastidas-Ramírez, Silvia Lucano-Landeros, Daniela Gordillo-Bastidas, Belinda C. Gómez-Meda, Arturo Santos, Eira Cerda-Reyes, Juan Armendariz-Borunda

**Affiliations:** 1 Institute for Molecular Biology in Medicine and Gene Therapy, Department of Molecular Biology and Genomics, Health Sciences University Center, University of Guadalajara, Guadalajara 44340, Jalisco, Mexico; 2 Laboratorio de Sistemas Biológicos, Centro Universitario de los Valles, Universidad de Guadalajara, Carretera Guadalajara-Ameca km. 45.5, Ameca 46600, Jalisco, Mexico; 3 Instituto de Investigación en Enfermedades Crónico Degenerativas, Department of Molecular Biology and Genomics, Health Sciences University Center, University of Guadalajara, Guadalajara 44340, Jalisco, Mexico; 4 Tecnologico de Monterrey, Escuela de Medicina y Ciencias de la Salud, Monterrey 64849, Nuevo Leon, Mexico; 5 Instituto de Genética Humana “Dr. Enrique Corona Rivera”, Department of Molecular Biology and Genomics, Health Sciences University Center, Guadalajara 44340, Jalisco, Mexico; 6 Hospital Central Militar, Mexico City 11600, Mexico

**Keywords:** liver fibrosis, muscle gene transfer, adenovirus vector

## Abstract

The development of several vaccines against the SARS-CoV2 virus and their application in millions of people have shown efficacy and safety in the transfer of genes to muscle turning this tissue into a protein-producing factory. Established advanced liver fibrosis, is characterized by replacement of hepatic parenchyma by tissue scar, mostly collagen type I, with increased profibrogenic and proinflammatory molecules gene expression. Matrix metalloproteinase 8 (MMP-8) is an interstitial collagen-degrading proenzyme acting preferentially on collagen type I when activated. This study was carried out to elucidate the effect of an intramuscularly delivered adenoviral vector containing proMMP-8 gene cDNA (AdhMMP8) in male Wistar rats with experimental advanced liver fibrosis induced by thioacetamide. Therapeutic effects were monitored after 1, 2, or 3 weeks of a single dose (3 × 10^11^ vp/kg) of AdhMMP8. Circulating and liver concentration of MMP-8 protein remained constant; hepatic fibrosis decreased up to 48%; proinflammatory and profibrogenic genes expression diminished: TNF-α 2.28-fold, IL-1 1.95-fold, Col 1A1 4-fold, TGF-β1 3-fold and CTGF 2-fold; and antifibrogenic genes expression raised, MMP-9 2.8-fold and MMP-1 10-fold. Our data proposes that the administration of AdhMMP8 in muscle is safe and effective in achieving liver fibrosis regression at a comparable extent as when the adenoviral vector is delivered systemically to reach the liver, using a minimally invasive procedure.

## 1. Introduction

At present, more than 30 anti-COVID-19 vaccines have been approved in the world (https://covid19.trackvaccines.org/vaccines/, (accessed on 30 April 2023)). At least four of the most used effective and safe vaccines are mRNA-1273, Moderna [1], BNT162b2, Pfizer/BioNTech [2], AZD1222, Oxford/AstraZeneca (Voysey 2021), Ad26.COV2.S, and Janssen [3], which are applied to muscle tissue turning it into a protein-producing factory. Currently, this strategy has become a widely used procedure in vaccine development. However, this methodology emerged approximately 30 years ago, and since then it has been used for the study of bacterial reporter gene expression in eukaryotic cells [4], production of hormones [5], and neurotrophic factors [6]. The safety in the application and the remarkable property of the muscle to take gene constructs up and express different types of proteins, led us to adapt this design for a secretory protein, matrix metalloproteinase-8 (MMP-8), through a model of experimental advanced liver fibrosis in rats.

Two million deaths per year in the world are attributable to liver diseases, and deaths in men due to these causes are up to 67%. Complications of cirrhosis and hepatocellular carcinoma originating from viral hepatitis, alcohol, and increasingly non-alcoholic liver disease, constitute the most common causes of death worldwide [7].

The advent of highly effective direct-acting antivirals has provided cure rates close to 100%, as an almost universal sustained virological response due to the elimination of HCV, has been achieved. Thereafter, the possibility to explore whether HCV eradication can reverse fibrosis and cirrhosis has emerged. With this regard, data are controversial since the extent of cirrhosis improvement and the type of patients that can experiment with this benefit is variable depending on individual drug response. However, hepatologists do agree that the quality of life of these patients is mainly due to the elimination of liver fibrosis and that the only current cure is liver transplantation. Therefore, the search for therapeutic strategies for the treatment of cirrhosis continues to be a primary goal [8,9,10,11,12].

Liver cirrhosis develops after a chronic period of liver damage leading to inflammation, that results in the replacement of the healthy liver parenchyma with fibrotic tissue generating regenerative nodules, and portal hypertension [13,14].

Liver fibrosis consists of an excessive deposition of extracellular matrix (ECM) induced by wound healing following liver damage, accompanied by inflammation and cell death [15].

In addition to the decreased activity of endogenous MMPs, hepatic fibrosis also shows a high expression of MMP tissue inhibitors (TIMPs), mainly TIMP-1 and 2. Thus, the combination of MMPs low activity, and TIMP high expression renders an ideal cellular environment to perpetuate the fibrogenic process by preventing degradation of fibrillar collagens [16,17].

**Figure 1:**
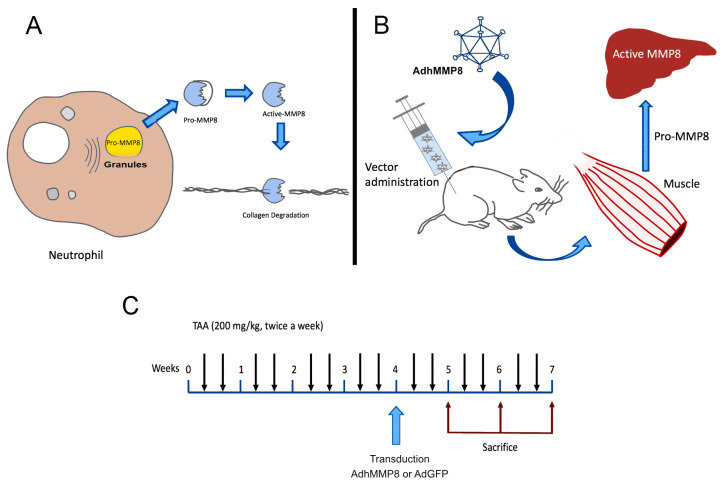
Study design. (**A**) MMP8 is produced by neutrophils, it is secreted as a pro-enzyme and activated degrading collagen; (**B**) the adenoviral vector AdhMMP8 is delivered intramuscularly to produce ProMMP8, becomes released systemically reaching the liver where it is activated to carry out its collagenolytic activity; (**C**) the adenoviral vector is administered 4 weeks after intoxication with TAA, animals continue receiving TAA intoxication and sacrificed after 5, 6, or 7 weeks (n = 5 rats/group).

The replacement of collagen IV by collagen I and II promotes deposition of ECM in the space of Disse, promoting the loss of sinusoidal endothelial fenestrations, and inducing a change in sinusoidal vascularization and damage to hepatocytes. Knowledge of these molecular events in the hepatic sinusoid has led to the implementation of gene therapy strategies for the treatment of liver fibrosis by trying to eliminate this ECM deposit [18]. ECM and connective tissue proteins are degraded by MMP-8, also known as neutrophil collagenase. The MMP-8 protein is translated as an inactive zymogen constituted by a prodomain that needs to be cleaved to become activated (Figure 1A), this domain is N-terminal with hemopexin-like repeats [19]. Substrates of MMP-8 mainly consist of fibrillar triple helix collagens type I, II, and III, fibronectin, gelatins, proteoglycans, and aggrecan, among others. MMP-8 in neutrophils is stored in intracellular granules as a proenzyme, and extracellular signals mediated by oxidative stress-derived molecules and other proteases, stimulate exocytosis at the site of the inflammation target [20].

The initial and rate-limiting enzymatic reaction to degrade interstitial collagen is mediated by MMP-8 [21]. Subsequently, the collagen fragments generated are broken down by gelatinases such as MMP-2, MMP-9 and, less importantly, by other MMPs, and serine proteases [22].

It has been described that regression of liver fibrosis is accompanied by a decrease of pro-inflammatory cytokines [interleukin 6 (IL-6), IL-1β, and transforming necrosis factor α (TNF-α)] and transforming growth factor β (TGF-β1) in the liver, and abrogation of ECM formation due to the production of collagen fibers-degrading MMPs [23]. Therefore, if we increase collagen degradation in the fibrotic liver using MMP8, it could be possible to trigger a process of liver regeneration.

Based on experimental protocols in animal models, fundamental changes regarding the possibility to regress hepatic fibrosis, were proposed in the late 90s [24]. In this field, our research group has contributed to the knowledge through several approaches using adenoviral (Ad) vectors [25,26,27,28], combinations of recombinant Ad vectors expressing different antifibrogenic genes [29]; recombinant Ad vectors plus stem cells [30]; and strategies to increase Ad vectors expression time [31]. However, acute hepatotoxicity in immunocompromised patients [32], and Ad side effects stimulating a strong inflammatory reaction in the liver causing hepatotoxicity [33], have largely limited the clinical application and driven the search for new approaches involving minimal invasion.

Thioacetamide (TAA) is the second most commonly used fibrosis-inducing hepatotoxin in rodents. TAA itself is nontoxic, but its metabolites, TAA sulfoxide, and TAA sulfdioxide, converted by CYP2E1, are hepatotoxic. The use of male rats instead of females is recommended to perform this experimental model since it is reported that TAA metabolism can be affected by hormonal variations. This model is suitable for the evaluation of fibrosis regression by treatment but inappropriate to investigate spontaneous regression [34].

The present study is unique in the field of gene therapy in liver diseases that occur with fibrosis because we are using a remote delivery strategy of an Ad vector that contains the human proMMP-8 gene to transduce muscle cells. This system is designed to convert muscle cells into producers of proMMP-8, secrete the protein systemically to carry out its collagenolytic activation in the fibrotic liver target (Figure 1B), and generate a change in the liver microenvironment, achieving a decrease in fibrosis without direct administration of the vector into the damaged target organ. Our goal is to provide the basis for the development of new strategies to revert hepatic fibrosis since as we mentioned before, not all patients respond in the same way to the same treatments. The possible availability of diverse treatments against liver fibrosis will be of great support to the millions of patients affected by this disease.

## 2. Materials and Methods

### 2.1. Adenoviral Vectors Production

Ad type 5 (Ad5) vector, carrying the green fluorescent protein (AdGFP) as a reporter gene, and human proMMP-8 cDNA (AdhMMP8), used for the transduction assay, were produced and amplified in our laboratory, as reported elsewhere. Briefly, recombinant adenovirus ΔE1 and ΔE3 serotype 5 AdhMMP8 and AdGFP were amplified in cultured HEK-293 cells (293 [HEK-293] CRL-1573 ATCC, USA), using DMEM media (Invitrogen, Grand Island, NY, USA) supplemented with 10% fetal bovine serum (Invitrogen, Grand Island, NY, USA), 37 °C and 5% CO_2_ atmosphere. Cells were collected after 48 h of Ad transduction and disrupted using three freeze/thawing cycles. Cellular debris was removed by centrifugation and supernatant containing Ad vectors was purified by ultracentrifugation at 141,000× *g* in a CsCl density gradient. AdhMMP8 and AdGFP particles were determined by optical densitometry [35]. Human proMMP-8 was chosen in our study in order to be able to distinguish between the endogenous expression and the transduced protein.

### 2.2. Animals and Experimental Design

Wistar rats used in this study were purchased from Charles Rivers (Boston, MA, USA) and maintained under the protocols established by the University of Guadalajara for Animal Care. Sixty male Wistar rats, 7–9 weeks old, weighing 200–250 g were divided into 4 groups (n = 15), as follows (Figure 1): Healthy, receiving no treatment; TAA, rats intoxicated with TAA delivered intraperitoneally twice a week during 7 weeks to develop liver fibrosis; TAA-AdGFP, rats intoxicated with TAA to develop liver fibrosis and treated with the adenovirus containing an irrelevant reporter gene (AdGFP); and TAA-AdMMP8, animals intoxicated with TAA and treated with the therapeutic gene (AdhMMP8). All groups were subdivided into three subgroups (n = 5): subgroups 1, 2, and 3 were sacrificed at the 5th, 6th, or 7th week of the experimental period, respectively. Both TAA-AdGFP and TAA-AdhMMP8 subgroups were intoxicated with TAA during 4 weeks followed by the intramuscular injection of the transduction vector, AdGFP or AdhMMP8, according to their corresponding group; subgroup 1 resumed TAA intoxication during one additional week before sacrifice at the 5th week of the experimental period; subgroup 2 continued TAA intoxication during 2 additional weeks before sacrifice at the 6th week; and subgroup 3 continued TAA intoxication during 3 additional weeks before sacrifice at the 7th week (Figure 1C).

### 2.3. TAA Liver Cirrhosis Model

TTA (Merck KGaA, Darmstadt, Germany) intoxication is an in vivo model used to analyze fibrosis prevention by AdhMMP8 treatment. TAA-induced cirrhosis was achieved using a dose of 200 mg/kg TAA intraperitoneally administered two times a week for 7 weeks as previously described [36,37,38].

### 2.4. Adenovirus Treatment in TAA-Intoxicated Rats

TAA-intoxicated rat groups were designed to assess the possibility that fibrosis progression could be improved. AdhMMP8 and AdGFP intramuscular delivery were carried out using a single dose of 3 × 10^11^ vp/kg in TAA-GFP and TAA-AdhMMP8 groups by injection in the biceps femoris muscles. Rats were sacrificed at the 5th, 6th, and 7th weeks, according to subgroups, as explained above. Immediately before sacrifice, blood samples for biochemical determinations were obtained. In both groups, representative liver sections were preserved in 4% paraformaldehyde for histological study, or frozen immediately for protein and RNA isolation, Also, in the TAA-GFP group, a muscle tissue sample was taken to observe the expression of the GFP reporter gene under a fluorescence stereoscope (Olympus SZX12) using a magnification of 50×.

### 2.5. Quantitative Real-Time Reverse Transcriptase (RT-PCR)

Total RNA was isolated according to Chomczynski and Sacchi’s modified method [39]. Briefly, liver tissue was homogenized in the presence of a Trizol reagent (Invitrogen, Carlsbad, CA, USA). Chloroform was added and the aqueous phase was isolated. RNA was precipitated with isopropanol. RNA quantity and quality were determined in NanoDrop equipment (Thermo Scientific, Hammonton, NJ, USA). Reverse transcription PCR using 2 µg of total RNA was carried out using M-MLV reverse transcriptase (Invitrogen, Carlsbad, CA, USA). Then, 2 µL of cDNAs were subjected to real-time PCR using a Rotor Gene thermocycler (Quiagen, 19300 Germantown Road, MD 20874, USA), under the following conditions: 2 min at 50 °C, 5 min at 94 °C, and 45 cycles of 30 s at 94 °C and 40 s at 60 °C. Taqman probes for COL1A1 (RN00670303_G1), CTGF (RN01537278_G1), TGF-β1 (RN00572010_M1), IL1-β (RN00580432_M1), TNF-α (PN99999017_M1), MMP-1 (LLC185875319), and MMP-9 (RN00579162_M1) rat cDNAs specific detection were bought from Applied Biosystems/Thermo Fisher Scientific (Hammonton, NJ, USA). Gene amplification was normalized against β-actin (RN00667869_M1) expression using the 2^−ΔΔCt^ method, measuring the ratio of specific gene expression showing the experimental treatment effect and the expression of a housekeeping internal control gene [40,41]. 

### 2.6. Histological Examination of Liver Sections

Livers were dissected and immersed in 4% paraformaldehyde (Merck KGaA, Darmstadt, Germany) diluted in PBS, dehydrated in alcohol, and embedded in paraffin. Light microscopy was utilized to evaluate sections 4 µm thick stained with Masson’s trichrome and Sirius Red by observing twenty random fields per slide and using a computer-assisted morphometric analyzer (Image-ProPlus 6.0; Media Cybernetics, Inc., Bethesda, MD, USA), as previously described [23]. Magnification was 200×.

### 2.7. Biochemical Assays

Animals were anesthetized using intramuscular 20 mg/kg Zoletil. Blood samples were collected through intracardiac puncture before sacrifice and centrifuged at 1500× *g*, 4 °C, for 10 min to obtain serum. Serum aliquots were stored at −80 °C. Serum levels of AST and ALT were quantified using a VITROS DT60II dry chemistry analyzer (Johnson & Johnson, Nuevo Brunswick, NJ, USA).

### 2.8. MMP-8 Protein Determination

Tissue proteins were extracted from 100 mg of liver tissue and minced in a buffer solution containing 65 mmol/L Tris, 310 mmol/L KCl, 1% Nonidet P40, 0.1% SDS, 200 mmol/L sodium orthovanadate, 1 mol/L NaF and a protease inhibitor cocktail (Roche Diagnostics, IN, USA). After centrifugation at 21,000 rcf/4 °C for 1 h, supernatant was stored at −80 °C. Protein quantification was performed by a Bradford assay. Serum and liver MMP-8 protein was quantified by ELISA (human total MMP-8, DY908 R&D systems, Minneapolis, MN, USA), a specific polyclonal antibody against human neutrophil MMP-8 was used, which showed no cross-reactivity with MMP-1, 2, 3, 7, 9, 10, 12 and 13 or MT1-MMP, and TIMP-1 and TIMP-2. Pro-MMP-8 and active MMP-8 were also detected by this ELISA system. This DuoSet was calibrated against a highly purified NS0-expressed recombinant human MMP-8 produced by R&D Systems. Levels of liver protein were normalized to 100 mg of liver tissue.

### 2.9. Statistical Analysis

Data are shown as the mean ± standard deviation (SD). For real-time PCR experiments, results are expressed as the 2^−ΔΔCt^ value (mean ± SD). Non-normally distributed data were analyzed using Mann Whitney U, Dunn’s, and Kruskal-Wallis tests, where statistical significance was considered when *p*-values were under 0.05.

## 3. Results

### 3.1. Adenoviral Vectors Amplification, Analysis of Muscle GFP Gene Expression, and Determination of Serum MMP-8 Protein

Both AdGFP and AdhMMP8 adenoviral vectors used in this study contained the CMV promoter and were similar in structure. Spectrophotometric titration of AdGFP and AdhMMP8 was 5.97 × 10^12^ vp/mL and 9.27 × 10^12^ vp/mL, respectively.

**Figure 2:**
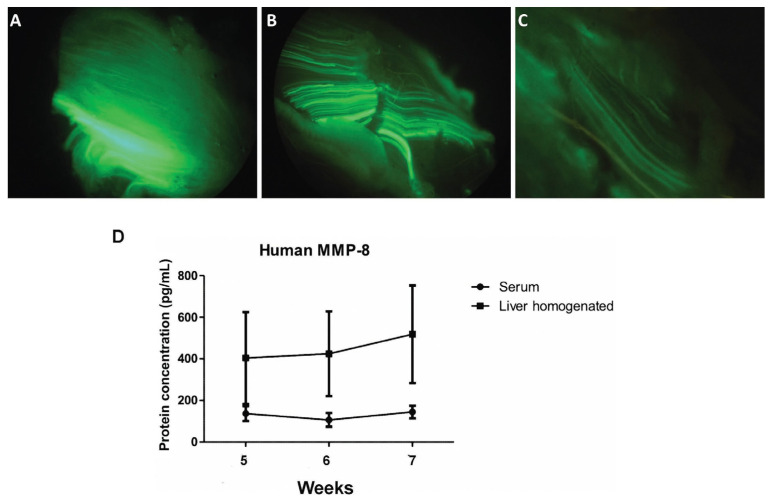
Expression of the adenoviral vectors (n = 5 rats/group). (**A**) GFP muscle fluorescence detection 1 week after the AdGFP vector transduction; (**B**) GFP muscle fluorescence detection 2 weeks after the AdGFP vector transduction; (**C**) GFP muscle fluorescence detection 3 weeks after the AdGFP vector transduction corresponding to 5, 6, and 7 weeks after TAA intoxication; (**D**) serum and liver human MMP8 levels, showing no significant differences among groups along the studied period. Liver protein levels were normalized to tissue weight (100 mg/sample).

To assess therapeutic protein expression, the AdGFP vector was administered to the TAA-AdGFP group, and AdhMMP8 to the TAA-AdhMMP8 group. As stated in the material and methods section, vectors were delivered at the end of the 4th week after TAA intoxication, continuing TAA intoxication for 1, 2, or 3 additional weeks before sacrifice, according to the experimental subgroup. GFP expression was observed in muscle tissue after administration of AdGFP, as shown in Figure 2A–C. Since the GFP protein is not a secreted but an intracellular protein, the fluorescence pattern observed reveals muscle transduction. These results indicate that AdGFP and AdhMMP8 vectors did not migrate to the liver or other organs, but remained circumscribed to the muscle where they were applied [42].

Quantification of human MMP-8 protein was performed by an ELISA assay on serum and liver tissue samples from the studied groups and was detected only in the TTA-AdhMMP8 group. The following amounts of human MMP-8 were found in serum: 137.0 ± 36.2 pg/mL, 106.5 ± 33.1 pg/mL and 144.9 ± 30.3 pg/mL; and liver samples from cognate animals: 404.2 ± 220.9 pg/mL, 424.3 ± 203.8 pg/mL and 518.5 ± 234.7 pg/mL, in the 5th, 6th, and 7th week, respectively, showing no statistically significant differences. Comparing the amount of serum and liver human MMP-8 protein, a 2.95-fold concentration in tissue at the 5th week (*p* < 0.01); 3.98-fold at the 6th week (*p* < 0.01); and 3.58-fold at the 7th week (*p* < 0.05), were observed (Figure 2D). Levels of MMP-8 protein in the liver were normalized to tissue weight (100 mg/sample).

### 3.2. Liver Function after AdhMMP8 Intramuscular Administration

Hepatic enzymes were evaluated to assess liver function improvement. Healthy rats showed 226.0 ± 61.2 IU/L and 64.7 ± 3.6 IU/L for AST and ALT respectively. TAA group showed 311.0 ± 112.1 IU/L and 182.4 ± 12.8 IU/L in the 5th week; 551.5 ± 76.4 IU/L and 171.6 ± 42.4 IU/L in the 6th week; and 270.0 ± 58.9 IU/L and 200.0 ± 183.8 IU/L in the 7th week for AST and ALT, respectively. TAA+AdGFP group showed 346.8 ± 185.8 IU/L and 195.6 ± 20.7 IU/L in the 5th week; 484.0 ± 164.8 IU/L and 172.8 ± 43.0 IU/L in the 6th week; and 276.8 ± 57.2 IU/L and 130.0 ± 47.4 IU/L in the 7th week for AST and ALT, correspondingly. TAA-AdhMMP8 group showed 214.0 ± 11.2 IU/L and 117.5 ± 46.8 IU/L in the 5th week; 320.5 ± 137.4 IU/L and 133.5 ± 25.7 in the 6th week; and 159.0 ± 26.6 IU/L and 89.0 ± 13.6 IU/L in the 7th week for AST and ALT, respectively. A significant difference (*p* < 0.05) in AST was observed when the TAA-AdhMMP8 group was compared with the TAA group in the 6th week (*p* < 0.05), as described in Table 1.

### 3.3. Liver Fibrosis Decreased after Intramuscular Injection of AdhMMP8

**Figure 3:**
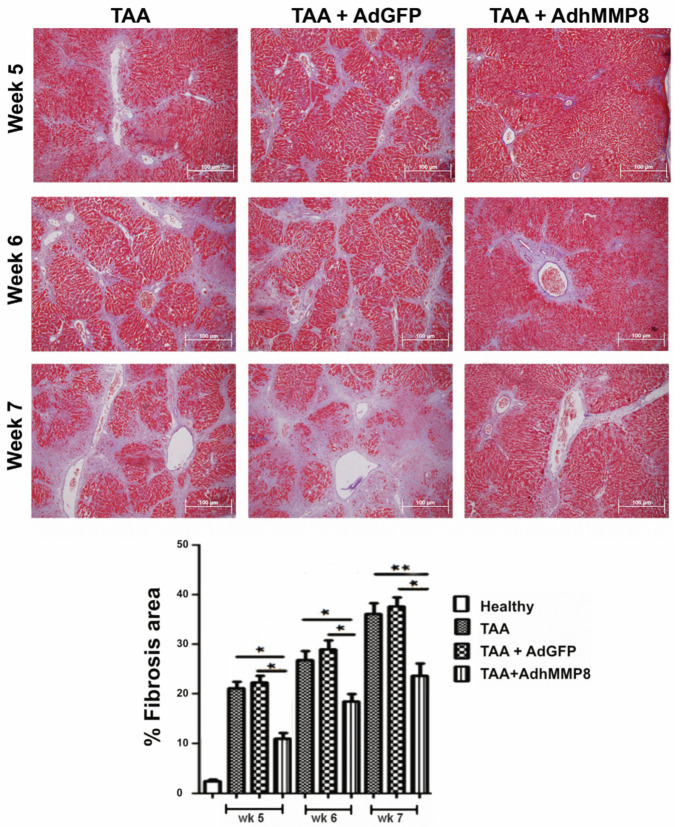
Analysis of liver fibrosis by Masson´s staining 5, 6, and 7 weeks after TAA intoxication, corresponding to 1, 2, and 3 weeks after the adenoviral transduction, using a computer-assisted morphometric method (Image-ProPlus 6.0; Media Cybernetics, Inc., Bethesda, MD, USA), and a magnification of 200×. Fibrotic bridges decreased up to 48% in the TAA + AdhMMP8 treated group when compared to TAA+AdGFP or TAA control groups (* *p* < 0.05; ** *p* < 0.01).

Hepatic fibrosis amount was determined through a computer-assisted morphometric method as a percentage of liver fibrous tissue (Image-ProPlus 6.0; Media Cybernetics, Inc., Bethesda, MD, USA) at a 200× magnification. Normal morphology exhibiting scarce ECM and radiated hepatocytes was observed in tissues from healthy rats. Altered morphology was observed in slides from TAA and TAA+AdGFP animals, where thick collagen septa forming bridges between portal tracts and central veins, were appreciated. In contrast, a higher hepatocyte proportion and lower ECM content in the TAA-AdhMMP8 group at the 5th, 6th, and 7th week of the experimental period, corresponding to 1, 2, or 3 weeks after AdhMMP8 delivery, respectively, was observed. Fibrosis measured in the healthy/control group was set at 2.7%; meanwhile, the TAA group exhibited 21.0%, 26.7%, and 36.0% after 5, 6, or 7 weeks of TAA intoxication, respectively. No significant differences between TAA and TAA+AdGFP groups were noticed. The percentage of fibrosis shown in the TAA + AdMMP-8 group was statistically lower when compared to the TAA group, with an average of 10.9%, 18.3%, and 23.4% after 5, 6, or 7 weeks, respectively. In other words, fibrosis in TAA + AdMMP-8 group decreased by 48.1% (*p* < 0.05), 32% (*p* < 0.05), and 45% (*p* < 0.01) during the studied periods, correspondingly (Figure 3).

**Figure 4:**
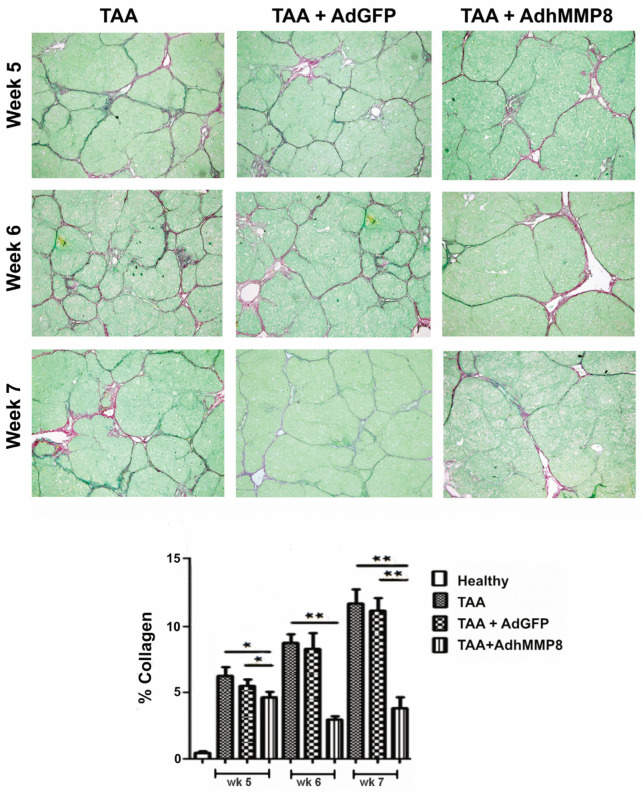
Liver collagen quantification by Sirius Red staining 5, 6, and 7 weeks after TAA intoxication, corresponding to 1, 2, and 3 weeks after the adenoviral vector transduction, using a computer-assisted morphometric method (Image-ProPlus 6.0; Media Cybernetics, Inc., Bethesda, MD, USA), and a magnification of 200×. Collagen fibers diminution of up to 65.5% can be appreciated in the AdhMMP8 vector-treated group when compared to the controls (* *p* < 0.05; ** *p* < 0.001).

When Sirius Red staining was performed for the analysis of collagen-specific quantification, the following fibrosis percentages were appreciated in the TAA group: 6.2%, 8.3%, and 11.6%, in the 5th, 6th, and 7th weeks, respectively. When comparing the percentage of fibrosis observed in the TAA and TAA + Ad-GFP groups, no significant differences were observed; therefore, comparisons were performed against only the TAA group. The fibrosis percentage observed in the TAA + AdhMMP8 group was lower when compared to the TAA group, with average values of 4.7%, 3.1%, and 4% in the 3 reference points, respectively, representing fibrosis reduction of 24.2% (*p* < 0.05) at the 5th week, 62.6% (*p* < 0.001) at the 6th week, and 65.5% (*p* < 0.001) at the 7th week (Figure 4).

### 3.4. Fibrogenic Molecules Gene Expression after Intramuscular Injection of AdhMMP8

**Figure 5:**
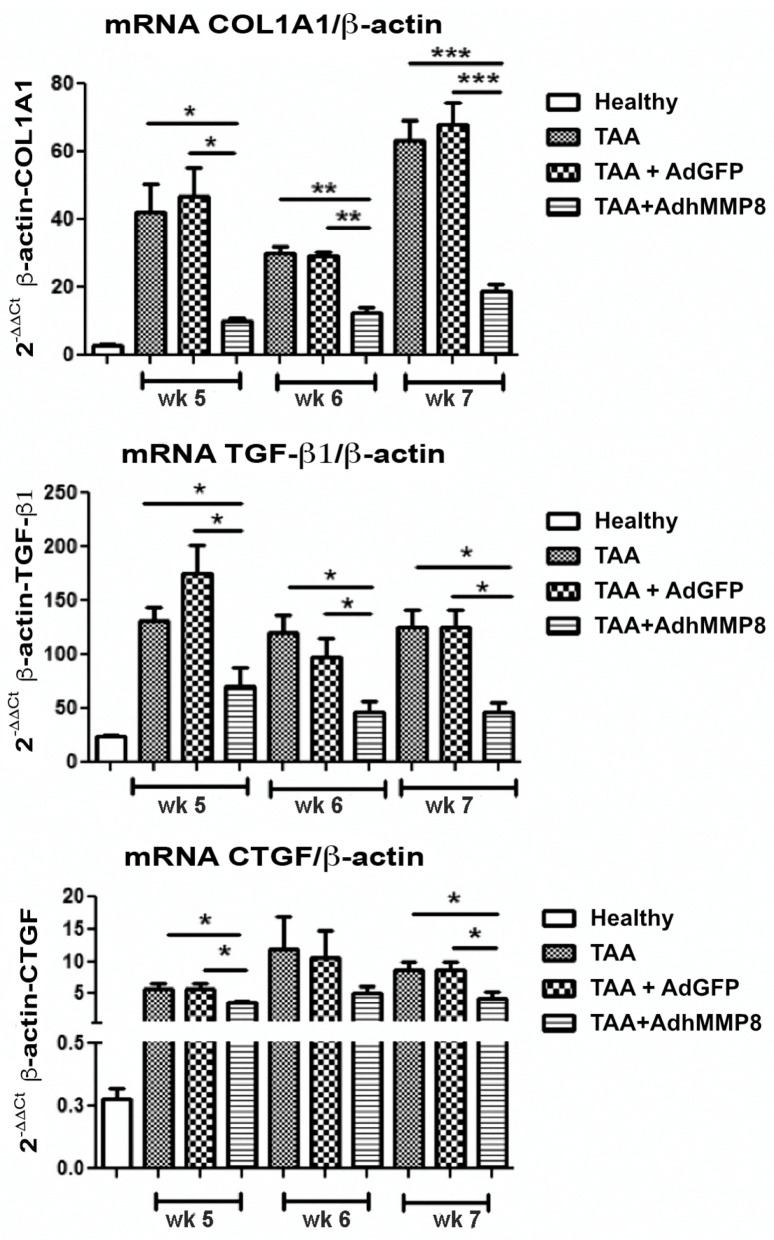
Profibrogenic genes mRNA level expression. A significant decrease of COL1A1, TGF-β1, and CTGF mRNA levels is shown in the AdhMMP8 vector-treated group when compared with the controls (* *p* < 0.05, ** *p* < 0.01, *** *p* < 0.001).

In addition to testing the antifibrogenic effect of AdhMMP8 at the histological level, mRNA expression of major genes promoting fibrosis COL1A1, TGF-β1, and CTGF, was analyzed in liver homogenates through real-time RT-PCR. Increased significant expression of all profibrogenic genes was appreciated in the TAA group when compared to the healthy group. No statistically significant differences were appreciated between TAA and TAA+AdGFP groups. TAA-AdhMMP8 animals presented 4.2 (*p* < 0.001), 2.45 (*p* < 0.05), and 3.37 (*p* < 0.05) fold decreased in COL1A1 gene expression after 5, 6, or 7 weeks of TAA intoxication, equivalent to 1, 2, or 3 weeks after AdhMMP8 transduction when compared to TAA group (Figure 5). Moreover, the TAA-AdhMMP8 group presented a 2.5 (*p* < 0.05), 2.9 (*p* < 0.05), and 3.5 (*p* < 0.05) fold lower expression of TGF-β1 at the 5th, 6th, and 7th week, respectively. Likewise, lower expression of the CTGF gene was observed in the TAA-AdhMMP8 group showing 2 (*p* < 0.05), 1.5 (NS) and 2.3 (*p* < 0.05) fold lower expression at the assayed periods when compared with the TAA group. No statistically significant differences were appreciated between TAA and TAA+AdGFP groups.

### 3.5. Antifibrogenic Molecules Gene Expression after Intramuscular Injection of AdhMMP8

Liver cell proliferation increment after the Ad delivery of MMP-8, indicating that MMP-8 in addition to promoting ECM remodeling also contributes to tissue regeneration, has been documented [32]. This process is possibly mediated by the release of growth factors associated with ECM, such as hepatocyte growth factor (HGF), the activation of other endogenous MMPs, and diverse components.

**Figure 6:**
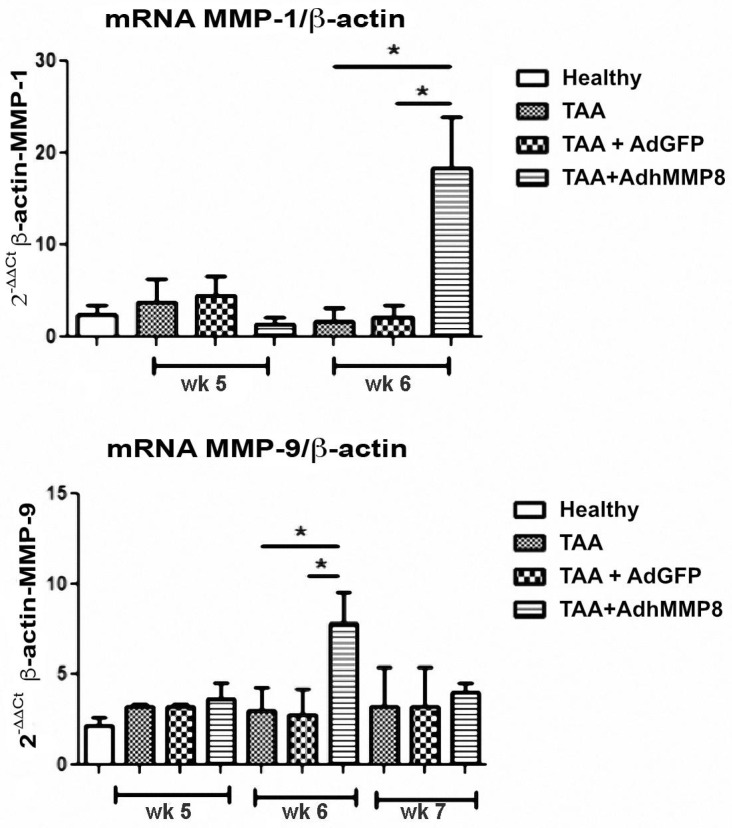
Antifibrogenic genes mRNA level expression in liver tissue. A significant increase of MMP-1 and MMP-9 mRNA level expression can be appreciated in the AdhMMP8 vector-treated group at week 6 when compared with the controls (* *p* < 0.05).

In this work, we evaluated two MMPs: mainly MMP-1, an interstitial collagenase; and MMP-9, a gelatinase. MMP-1 gene expression presented a significant (*p* < 0.05) 10.8-fold increase in the TAA + AdMMP8 group in the 6th week when compared to the TAA group; meanwhile, MMP-9 gelatinase showed an increase in TAA-AdhMMP8 group at the three reference studied points, reaching a significant (*p* < 0.05) 2.8-fold increase at the 6th week (Figure 6).

### 3.6. Inflammatory Gene Expression after Transduction with AdMMP8

**Figure 7:**
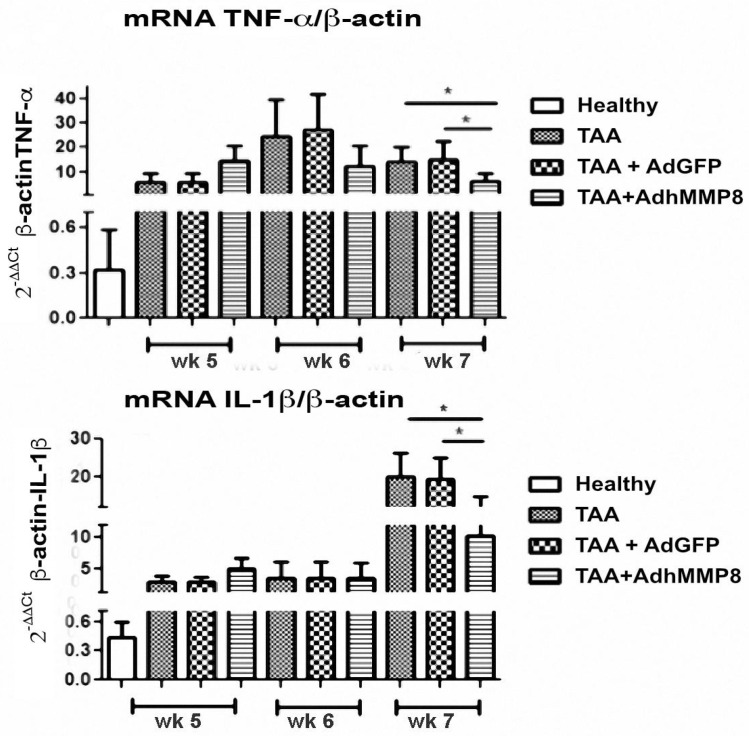
Proinflammatory genes mRNA level expression in liver tissue. A significant decrease in TNF-α and IL-1β mRNA level expression can be observed in the AdhMMP8 vector-treated group at week 7 when compared with the controls (* *p* < 0.05).

Increased TNF-α gene expression in the acute stages of the fibrotic process was observed in the TAA-AdMMP8 group in the 5th week in comparison with TAA and TAA+AdGFP groups. Meanwhile, a subsequent statistically significant (*p* < 0.05) gene expression diminution of 2.28-fold in the 7th week was exhibited (Figure 6). Similarly, IL-1β gene expression, an important proinflammatory gene, presented a significant (*p* < 0.05) diminution of 1.95-fold in the TAA-AdMMP8 group in the 7th week, when compared with the TAA group (Figure 7).

## 4. Discussion

According to data from the CDC (Centers for Disease Control and Prevention), approximately 670 million doses of vaccines to prevent COVID-19 using the intramuscular route, have been administered up to April 2023 in the United States of America (https://covid.cdc.gov/covid-data-tracker/#vaccinations_vacc-total-admin-rate-total, (accessed on 30 April 2023)).

CanSino and Sputnik anti-SARS-CoV2 vaccines, using Ad5 in their manufacturing, have been delivered to the population of at least 20 countries, providing evidence of systemic viral S protein production (https://covid19.trackvaccines.org/vaccines/, (accessed on 30 April 2023)). These facts, demonstrate the feasibility that the muscle has the capability to become an efficient protein-producing tissue. Similar biotechnological development is proposed in this work for liver fibrosis reversion through the over-expression of the human proMMP-8, since its antifibrogenic activity in the reversal of fibrosis [36], and its activation restricted to the site of inflammation [19], has been documented. The goal of this work was to promote muscle proMMP-8 systemic secretion, enzyme activation in the liver, and finally accomplishment of liver fibrosis regression. Our strategy was based on two major points: the use of skeletal muscle as a protein producer of secretory proteins, and the transfection of proMMP-8 cDNA through Ad5.

The involvement of MMP-8 in a wide variety of inflammatory disorders have been evidenced in diverse studies, turning MMPs activation into a tightly regulated mechanism. MMP-8, translated as an inactive proenzyme as most MMPs, contains a cysteine residue in its N-terminal pro-domain which participates in the blockage of its proteolytic activity by interaction with the Zn^2+^ ion at the active site. MMP-8 activation can be accomplished by disruption of this system, known as the cysteine-switch mechanism, either by proteolytical remotion of the pro-domain or by cysteine thiol group modification. Reactive oxygen species from activated neutrophils, as well as other proteases, promote the conversion of MMP-8 proform into an active protease. Reactive oxygen species released from activated neutrophils or a variety of proteases acting at the site of inflammation might mediate this change. Some MMPs, such as MMP-3, MMP-7, MMP-10, and MMP-14, or proteins such as cathepsin G and chymotrypsin, and even several proteases from bacteria, can activate MMP-8. In this way, the activation of MMP-8 as a tightly regulated process being accomplished at the site of inflammation, has been clearly evidenced. Active MMP-8 is not only a potent protease that can degrade mainly collagen type I, followed by collagen type III and type II; but it can also degrade substrates different from collagen, such as ECM. As ECM functions as a barrier between hepatocytes and blood vessels, its breakdown can lead to the release of many associated factors allowing a signaling cascade of anti-fibrogenic proteins.

The results obtained in this work, demonstrate the potential of AdhMMP8 vector to efficiently transduce skeletal muscle cells, resulting in a sustained expression of MMP-8 protein or GFP, during at least 21 days. The presence of the protein was evidenced from the 5th to the 7th week of the study through ELISA detection. Serum human MMP-8 levels were present at a steady level at the three experimental points evaluated after muscle transduction, showing an average of 129.3 ± 33.2 pg/mL. When this protein was quantified in liver tissue homogenates from cognate rats, average higher levels were observed, 448.6 ± 219.8 pg/mL/100 mg protein, equivalent to approximately 3.5-fold times the amount detected in serum at the corresponding treatment period.

The intramuscular route was chosen due to the easy access and the large surface area available for gene expression and delivery. If only some cells were transduced, the translated protein would be secreted into the adjacent muscle and/or the circulation, reaching remote tissues, such as the liver [43], and then activated by several proteases, such as plasmin [18], stromielysin [44], reactive oxygen species, and other MMPs [45].

AdMMP8 delivered into the skeletal muscle of rats resulted in a considerable amount of circulating and liver tissue protein during a period of three weeks, histological fibrosis and proinflammatory molecules expression lowering, and MMPs activity increase, all key components of the fibrogenic process remodeling.

To validate the transduction achieved by the Ad5 vector, the presence of GFP in skeletal muscle was evaluated after the administration of 3.0 × 10^11^ vp/kg. The presence of GFP was monitored by fluorescence microscopy for up to 21 days. Fluorescence was exclusively detected in the muscle where the administration was carried out, but not in the liver, lung, heart, spleen, kidney, or other organs, providing evidence of the systemic expression of the protein lacking the transduction to other organs. These results coincide with those reported by Tripathy [46], who determined the Ad5 genome by PCR, and concluded that Ad5 at a dose of 1 × 10^7^ to 1 × 10^9^ pfu delivered through an intramuscular route, was kept localized at the injection site and excluded the possibility to cause infections in injected neonatal mice. Moreover, they also detected Ad5 only in the injected muscle, but not in the brain, lung, heart, spleen, liver, gonads, or other organs of the experimental animals.

Diverse MMPs can degrade fibrillar-type collagen, as in liver fibrosis, but MMP-8, MMP-1, and MMP-13 have proven to display the main activity [47]. Collagenase delivery in advanced liver fibrosis using experimental models of liver cirrhosis is a strategy previously reported by our working team [32,34] and other research groups [48].

In order to eliminate the experimental caveat of spontaneous regeneration in established liver fibrosis in rats, a cDNA encoding human proMMP-8 was delivered in muscle concomitantly with chronic TAA intoxication. In this experiment, we tested the hypothesis that it is possible that MMP-8 gene transduced muscle can produce the protein, and then this protein systemically reaches and becomes activated in the liver, promoting an increase of ECM degradation in a TAA-fibrosis-induced model. It has been reported that rats exposed to TAA intoxication results in liver lesions resembling cirrhosis patterns or micronodular cirrhosis developed in humans [49]. During the TAA cirrhosis induction process, a reproducible temporary course of biochemical and morphological changes already described and defined in other publications [50,51,52], were also observed. Following TAA withdrawal, damage prevails for at least 2 months. Liver enzymes AST and ALT values exhibited no significant difference among groups throughout the course of the experiment, being AST significantly lower in the AdhMMP8 group in comparison to the TAA group only in the 6th week, equivalent to 2 weeks after the therapeutical gene delivery. This poor recovery of liver function was probably attributed to TAA-sustained intoxication. Upon observing TNF-α and IL-1β gene expression, a significant decrease of these proinflammatory molecules was appreciated in the 7th week, corresponding to 3 weeks after AdhMMP8 injection. A tendency to decrease liver enzymes and pro-inflammatory proteins was observed as well.

ECM deposit remodeling is performed through the induction of a positive balance by the presence of transgenic human MMP-8 upon activation within the cirrhotic liver. Fibrosis measured by morphometric analysis in slides stained with Masson’s trichrome after 3 weeks of AdhMMP8 administration, shows almost 50% diminution even when TAA intoxication was continued when compared with TAA-intoxicated animals with no treatment.

Furthermore, a decreased expression of COL1A1, TGF-β1, and CTGF, pro-fibrogenic genes, in addition to endogenous MMP-1 and MMP-9 activity upregulation after AdhMMP8 treatment, was demonstrated. However, other possible fibrinolytic processes may also be occurring. Diminution of MMPs expression constitutes a very important issue in the pathogenesis of advanced hepatic fibrosis and cirrhosis. Moreover, another control level over MMP’s activity is exerted by TIMPs, MMP’s physiological inhibitors, through proenzyme stabilization and inhibition of the active species [53,54]. Liver restoration has been observed when collagen 1 decreases, conducting to hepatic stellate cells (HSC) apoptosis and hepatocyte regeneration since collagen I influences the activation of HSC [55]. 

All our results suggest that the proMMP-8 enzyme, the protein overexpressed in muscle, has antifibrogenic activity in the liver. This is in accordance with a study demonstrating similar efficiency of pro-MMP8 and active MMP-8 sent directly to the liver, to reverse liver fibrosis utilizing Ad-Hepatitis B chimeric vectors [56].

It is worth mentioning that a single dose of AdhMMP8 in skeletal muscle resulted in the expression of serum MMP-8 at least for the three weeks monitored in our study, significantly reducing hepatic fibrogenesis, and showing no apparent systemic side effects. Importantly, fibrosis inhibition achieved correlates with the increase in tissue and circulating MMP-8. The results indicate the effectiveness of this strategy, as the expression of MMP-8 in a distant area from the site of administration can decrease liver fibrosis. Moreover, the use of AdhMMP8 in this way, facilitates the understanding of MMPs role in the organ where genetic transfer in a direct way may be limited or inhibited.

As it is known, limitations of systemic gene transfer utilizing Ad5 for gene therapy applications, due to the pre-existence of neutralizing antibodies. Depending on the geographical area or the economic situation, Ad5-neutralizing antibodies may be present in up to 80% of the population [57,58]. The health urgency caused by SARS-CoV2, which causes COVID-19 disease, has generated a lot of knowledge. CanSino and Sputnik V vaccines are based on an Ad5 vector to produce SARS-CoV2 viral proteins when applied in muscle tissue. Remarkably, regardless of the presence of neutralizing antibodies against the adenoviral vector, these vaccines have displayed an efficacy of 75–95% [59,60], indicating that the intramuscular route of administration plays an important role in gene transfer protocols.

The main goal of our study was to present evidence of fibrosis improvement by transducing muscle cells instead of liver cells, to produce human proMMP-8 protein, and secrete the protein systemically until reaching the damaged liver target. This is the first experimental gene therapy study using remote gene delivery to improve liver fibrosis.

## 5. Conclusions

We provided evidence of the effectiveness of remote gene transduction in ameliorating liver fibrosis, using this minimal invasive strategy that could be considered for further utilization in clinical trials.

## Figures and Tables

**Table 1:** Hepatic function tests (IU/L). Data represent the mean value ± SD (* *p* < 0.05).

Study Group (n = 15)	Time after TAA Intoxication					
	Week 5 (n = 5)		Week 6 (n = 5)		Week 7 (n = 5)	
	AST	ALT	AST	ALT	AST	ALT
**Healthy**	226.0 ± 61.2	64.7 ± 3.5	226.0 ± 61.2	64.7 ± 3.6	226.0 ± 61.2	64.7 ± 3.6
**TAA**	311.0 ± 112.1	182.4 ± 12.8	551.5 ± 76.4	171.6 ± 42.4	270.0 ± 58.9	200.0 ± 183.8
**TAA + AdGFP**	346.8 ± 185.8	195.6 ± 20.7	484.0 ± 164.8	172.8 ± 43.0	276.8 ± 57.2	130.0 ± 47.4
**TAA + AdhMMP8**	214.0 ± 11.2	117.5 ± 46.8	320.5 ± 137.4 *	133.5 ± 25.7	159.0 ± 26.6	89.0 ± 13.6

## Data Availability

Not applicable.
